# Easily misdiagnosed intramuscular hemangioma: a case report and literature review

**DOI:** 10.3389/fsurg.2025.1533174

**Published:** 2025-06-03

**Authors:** Rong Lei, Linqiang Tian

**Affiliations:** The Third Affiliated Hospital of Xinxiang Medical University, Xinxiang, China

**Keywords:** intramuscular hemangioma, gluteus medius muscle, misdiagnosis, vascular tumor, benign, case report

## Abstract

Intramuscular hemangioma is a benign tumor, usually occurring in the lower limbs. We report a rare case of intramuscular hemangioma that occurred in the gluteus medius muscle and was misdiagnosed as an injury to the gluteus medius muscle with bleeding. A 36 year old male presented with left hip pain after a clear history of trauma. He was diagnosed with damage to the gluteus medius muscle and bleeding, and received treatment, but the pain did not improve significantly. Magnetic resonance imaging(MRI) shows the formation of hematoma in the gluteus medius muscle.The patient underwent MRI enhancement and was ultimately diagnosed with a gluteal muscle hemangioma.Patients with intramuscular hemangioma usually do not have specific symptoms and have a clear history of trauma for this patient.Therefore, this type of tumor is often misdiagnosed. When the treatment effect is not ideal, the diagnosis should be re evaluated in a timely manner.

## Case report

**Figure 1:**
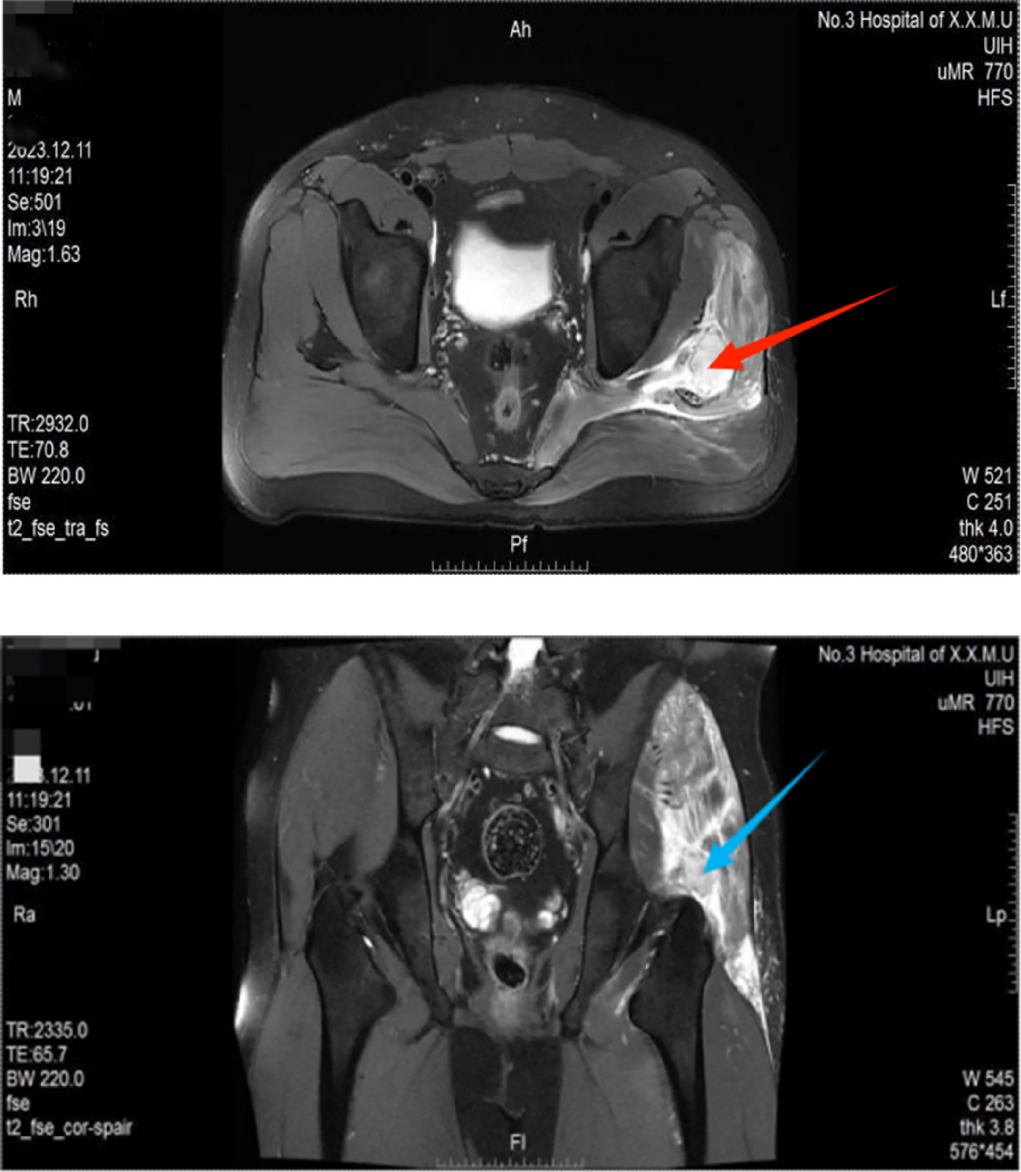
Upper Pic: Abnormal high signal shadow of the left gluteus medius muscle seen in MRI t2_fse_tra_fs (red arrow). Lower Pic: An abnormal enhancement confined to the gluteus medius muscle in MRI t2_fse_cor-spair (blue arrow).

A 36 year old male, first visited on December 11, 2023, complained of left hip pain after falling while playing ball a week ago. Physical examination: The left lower limb was about 0.5 cm shorter compared to the right one. Tenderness was noted at the greater trochanter in the left hip femur, along with a positive “4-word” test. The MRI (Figure 1) of the hip joint shows a hematoma in the left gluteus medius muscle (38.15 mm × 26.94 mm). Rest, decrease weight bearing, and seek symptomatic treatment with medication are advised. After one week of treatment, there was no significant improvement in the pain symptoms. The second visit took place on 2023-12-18, with the same examination as before. The patient was asked to take oral non-steroidal anti-inflammatory drug Etoricoxib, and continue resting, reducing weight bearing, and taking medication for symptomatic treatment. After 3 days of oral medication, the symptoms were still not relieved. The third visit occured on 2023-12-21, with the same physical examination as before, and intravenous infusion of sodium heptapodophylloside was recommended, and the pain symptoms still did not improve after the medication.

After that, the patient went to other hospitals, and through a phone call to the patient, we learned that the patient was finally diagnosed with “gluteus medius muscle hemangioma” through MRI enhancement examination in other hospitals.

We reviewed the treatment process of the patient, who came to the hospital for trauma and underwent an MRI examination. Combined with the patient's chief complaint, specialized examination, and current medical history, a diagnosis of gluteus medius muscle injury with hemorrhage was given, and we provided the patient with a treatment plan that reduced swelling, relieved pain, and recommended rest, but the patient's pain symptoms did not show significant improvement. At this point, we should consider whether the previous diagnosis was correct or not. The patient continued treatment at another hospital and underwent surgical treatment. The postoperative pathological diagnosis was that the gluteus medius muscle vessel was present. However, the patient refused to provide pathological photos, so only a written description could be provided. The pathological results from an external hospital showed that under the microscope, there were cell rich capillaries infiltrating the skeletal muscle tissue, accompanied by a small amount of mature adipose tissue and a small number of lymphatic vessels. Based on the histological morphology, the final diagnosis was intramuscular hemangioma, capillary type.

## Discussion

Hemangiomas can occur almost anywhere in the body, including the skin, subcutaneous tissue, muscle, visceral tissue, and bone. Intramuscular hemangiomas account for <0.8% of all hemangiomas (1, 2). Hemangiomas occur as a result of proliferation of vascular cells during the embryonic period, resulting in congenital benign tumors or vascular malformations within the skin and soft tissues.Intramyocardial hemangiomas account for less than 1% of all hemangiomas, and intramyocardial hemangiomas are benign vascular tumors of the skeletal muscle and are the most common type of deep soft tissue tumors (3, 4). Based on the cases reported so far, intramuscular hemangiomas can occur in any skeletal muscle and usually invade only one muscle, but about half of them occur in the lower extremities, with the thigh being the most common site (5). Hemangiomas of gluteal-related muscles are rare, and related reports in the literature are scarce.

The etiology of intramuscular hemangiomas is unknown, and by reading the literature, trauma and hormonal influences may be responsible for the etiology of the tumors or growth surges (6).

Early stage intramuscular hemangiomas have no specific symptoms (i.e., usually no mass, pain, or swelling). In later stages, patients are at risk of developing myosclerosis, contracture, muscle and joint deformities, and dysfunction, and their later symptoms mainly arise from the compression of adjacent muscle tissue, blood vessels, and nerves by the enlarged hemangioma (7). The insidious onset and slow progression of some cases make it difficult to confirm the diagnosis, and there are no obvious specific symptoms and signs in the early stage of intramuscular hemangiomas, which is an important reason why intramuscular hemangiomas are easy to be underdiagnosed and misdiagnosed (5, 8–10). Other reasons why this disease is often misdiagnosed include: ① the symptoms are not persistent, occurring intermittently, and are not emphasized by the patient. ② The lesions are deep, and there are often no obvious signs such as lumps or skin color changes on the body surface, even though professional doctors often cannot find lumps after careful physical examination. ③ Low incidence, the doctor lacks experience.

The possibility of intramuscular hemangioma should be considered when the following conditions are encountered clinically: ① limited soreness in the extremities in adolescents, and 80% to 90% of intramuscular hemangiomas occur before the age of 30 years (10). ② Fixed location, intermittent occurrence, commonly seen after exercise, considered due to increased blood flow in the affected muscle compressing nerves. Asymptomatic during the interval period. About half of them are located in the lower limbs, with the thighs being the most common (11). ③ x-rays show vein stones. Phleboliths are a more specific radiographic manifestation of hemangiomas and vascular malformations, which occur when intravascular thrombus calcifies. It occurs in about 25% of cases and is of greater diagnostic significance. ④ Ultrasound and MRI reveal deep soft tissue tumors. High-frequency ultrasound can be the noninvasive test of choice for intramuscular hemangiomas (6), and its concordance with pathologic diagnosis is 87%. It is recommended to routinely perform color ultrasonography when a mass is found, and if abnormal blood flow images are found, then further examinations such as MRI are performed. MRI is recognized as having the most diagnostic value for soft tissue hemangiomas (12), and it can clearly show the size of the mass, its borders, and the degree of involvement of the surrounding tissues, and the specific images of vascular diseases by MRI have important diagnostic value for masses of unknown nature in deep tissues. It has important value for surgical program and assessment of prognosis.

As there are no specific symptoms and signs in the early stage of hemangioma, it is easy to be misdiagnosed. In the case of “gluteus medius hemangioma”, it needs to be differentiated from the gluteus medius muscle injury caused by trauma; in the late stage, it can be misdiagnosed as “lumbar disc herniation” (5); in addition, it needs to be differentiated from the corresponding soft tissue malignant tumors. “Lumbar disc herniation” (5); Once the diagnosis of intramuscular hemangioma is confirmed, surgical resection is the better treatment modality, which requires surgical removal of the tumor or even the entire muscle, and the prognosis is better. When a patient is not a suitable candidate for surgery, sclerotherapy or combination treatment could also be considered (2, 13, 14).

Since intramuscular hemangiomas of the gluteus medius muscle are very rare, the pathogenesis, risk factors, recurrence rate, and complications of intramuscular hemangiomas of the gluteus medius muscle need further study.

## Data Availability

The original contributions presented in the study are included in the article/Supplementary Material, further inquiries can be directed to the corresponding author.
